# Date fruit detection dataset for automatic harvesting

**DOI:** 10.1016/j.dib.2023.109876

**Published:** 2023-11-30

**Authors:** Yousra Zarouit, Hassan Zekkouri, Mohamed Ouhda, Brahim Aksasse

**Affiliations:** a MIS Team, MAIS Laboratory, Department of Computer Science, Faculty of Sciences and Technics, Moulay Ismail University, BP 509 Boutalamine, Errachidia 52000, Morocco; b TIAD Laboratory, Department of Computer and Mathematics, Higher School of Technology, Sultan Moulay Slimane University, Khenifra, Morocco; c MAIS Laboratory, Department of Computer Science, Faculty of Sciences, Moulay Ismail University Meknes, Morocco

**Keywords:** Date fruits, Intelligent harvesting, Fruit classification, Fruit detection

## Abstract

Date fruit is one of the most beneficial and delicious fruits in the world through its nutritional value, it's a rich source of sugar, Protein, Manganese, Fibers, and many other vitamins, on the other hand, date production in the world reached up to 9.45 million tons, one palm tree can produce dates for 40–50 years which makes date production one of the main pillars of the economy. Generally, the methods used for pre and post-harvesting are done manually, making the operation heavy in terms of time and economy. Research in the area of harvesting automation is still limited due to the absence of data. This work presents a full dataset for detection, classification, analysis, and harvesting decisions. The dataset contains a collection of images and videos for different varieties of dates at different stages of maturity, taking into consideration all the natural conditions including light, contrast, dates in bags, and multi-scaled images. The big advantage of this dataset is it can help to put agriculture research at an advanced level by robotizing all the pre and post-harvesting tasks. The dataset is published for free in ZENODO [1] https://zenodo.org/doi/10.5281/zenodo.8315234.

Specifications Table

**Table:** 

Subject	Computer science, machine intelligence
Specific subject area	Object detection, image classification, automated harvesting, robotic
Data format	Raw data
Type of data	Images (.JPG) Videos (.MP4) Texts(.TXT)
Data collection	The dataset comprises 9092 images of date grapes, captured at various scales and angles between June and September 2022. The data collection occurred during the hours of 6 am to 6 pm, ensuring adherence to natural daylight conditions in a real-world environment. The images were obtained using both a Canon camera and a Samsung Ultra smartphone camera.
Data source location	University Moulay Ismail faculty of sciences and technology Errachidia, Morocco. the first orchard is located 26 km southeast of Errachidia, Morocco (31.8987333, -4.1997700)and the second is in Tismoumine, Alnif tinghir, Morocco (30.689038927052874, -5.126078230952724)
Data accessibility	Repository name: ZENODO Data identification number: 10.5281/zenodo.8315234. Direct URL to data: https://doi.org/10.5281/zenodo.8315235

## 1 Value of the Data

• Train object detection models to recognize and locate date fruit in an orchard. This can enable robots to autonomously navigate through the orchard and identify the fruit that is ready for harvest.
• The date fruit dataset is highly valuable for the future of robotics in agriculture. With the help of machine learning algorithms and computer vision, robots can be trained to identify and classify date fruits at different stages of maturity, which can then be used for a variety of tasks, such as date fruit detection and segmentation, automated harvesting, maturity analyses, quality control, and yield estimation.
• Improving yield estimation by using machine learning algorithms to analyse the dataset of date fruit, researchers can create models that accurately estimate the yield of the upcoming harvest. This can help growers make more informed decisions about when to harvest and how much to expect and can enable robots to plan and execute harvesting tasks more efficiently.
• The dataset contains various types of data such as image annotations, images, and videos taken under all daylight conditions which is essential to build a robust machine vision system.

## 2 Background

The current fruit dataset [1], [2] predominantly comprises images taken on a single date against a consistent background. This limitation hinders the development of a reliable automatic harvesting system capable of detecting and analyzing date fruit within an orchard. In contrast, a recent dataset [3] has emerged, featuring images of five popular date varieties in Saudi Arabia captured in a natural environment, specifically designed for automatic harvesting. Notably, the proposed dataset offers a distinct advantage, as it goes beyond simple classification. It encompasses both classification and object detection, incorporating four prevalent date varieties in Morocco. This endeavor introduces a comprehensive dataset that addresses detection, classification, analysis, and decision-making for harvesting processes.

## 3 Data Description

The date fruit dataset for automatic harvesting can serve several purposes, including all harvesting stages such as date detection and classification, maturity analysis, and the harvesting decision. The dataset contains 9096 images of 128 bunches of dates for different Moroccan varieties of dates covering all the maturity stages. Moreover, the dataset is characterized by images from different angles under various conditions such as illumination, dates covered by bags, and dates hidden by palm leaves. Date fruit dataset can provide a valuable resource for the development of robotic systems in agriculture, enabling more efficient and sustainable production of crops [4,5]. Table 1 provides a summary of the characteristics of the dataset.

**Table 1:** Overview of dataset characteristics.

Characteristics	Description
Number of dataset Images	9092 images of date bunches.
Type of data	Images (JPG), videos(MP4), text file
Varieties of dates	Majhoul, Boufaguos, Kholt, and Bouisthami
Maturity stages	Immature, Khalal, Rutab, and Tamar stage
Dataset label and annotation	The Dataset labeled to classify and annotated to detect date fruit based on both their varieties and maturity stages.
Images variation	Images multi-scale, variable daylight illumination, and dates hidden by bags and palm leaves.

**Fig. 1:**
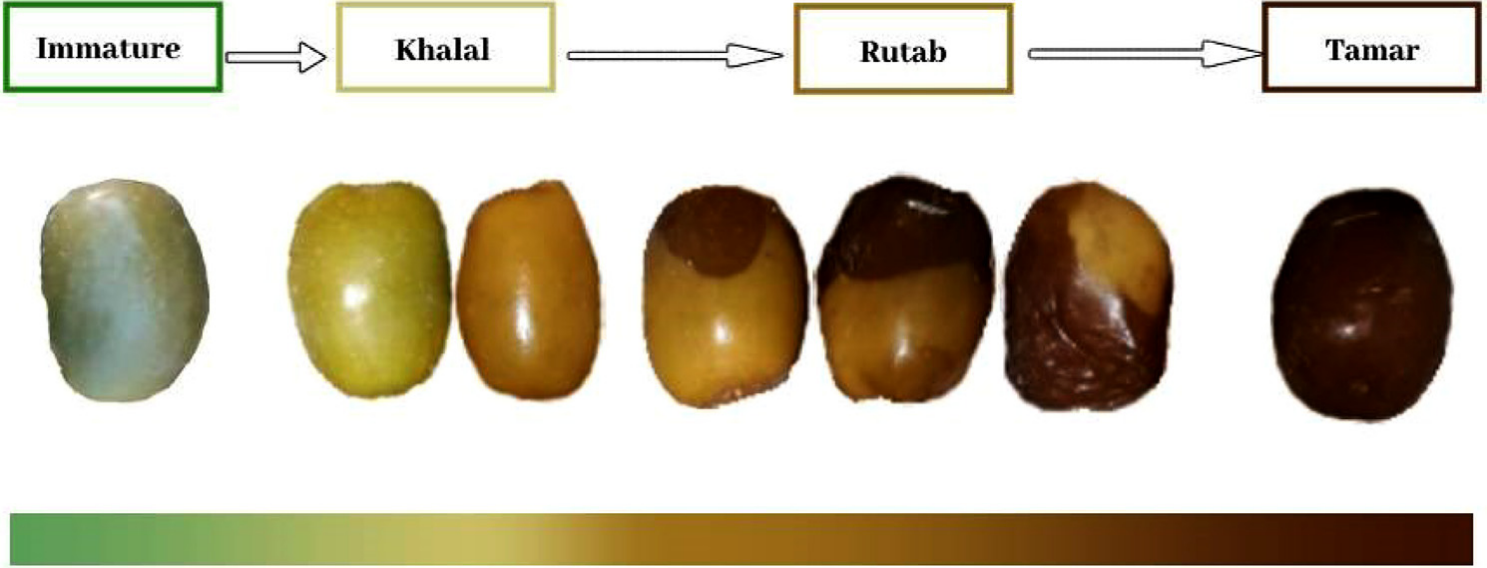
Date fruit maturity stages.

The development of date fruit pass through several maturity stages before they are entirely harvested. The maturity stages of date fruit are classified into four classes: Immature, Khalal, Rutab, and Tamar, as shown in Fig. 1.

The first stage in the maturity process is the immature stage, also known as Kimri. This stage usually lasts around 8-14 weeks, depending on the variety and climatic conditions. Kimri is the longest stage of growth of dates. The fruit in this stage is characterized by its green color, hard texture, and rapid growth in weight and size. Generally, the dates in this stage are not ready to harvest.

The Khalal stage is the second stage of growth. Dates in this stage change color from green to greenish-yellow, yellow, and red, depending on the variety. This stage usually lasts for around 3–5 weeks. Dates in this stage reach their maximum size and weight with a firm texture and be ready for the first season of harvesting to be consumed as fresh ripe fruit.

The Rutab stage starts when dates start to take brown or black color gradually from the sides to the whole fruit and become soft. This stage takes around 2–4 weeks. Rutab is the best phase to store the dates in cold stores. Otherwise, if it is not kept in the fridge after harvest, its taste changes and becomes inconsumable.

The Tamar stage is the final phase of maturity following the Rutab stage. Dates color becomes dark brown or black, and the texture is soft with a slight stickiness. The date fruit in this stage loses moisture content, and the sugar content increases, making it sweeter and non-perishable it can be conserved for a long time.

In summary, Harvesting decisions depend on date variety, conservation, and climatic condition.

To create a robust harvesting system that can replace humans in identifying, classifying, and harvesting ripe fruit, the system must overcome some key challenges facing machine learning. Therefore the dataset should contain all the natural conditions of date. The dataset includes 9096 images covering several variations. Each of the following variations plays a critical role to create an automatic harvesting system that can accurately identify and locate ripe fruit under various conditions, leading to more efficient and profitable harvesting operations.

• Different date fruit varieties:

There are more than 200 types of date fruit that have different sizes, shapes, and colors. Training a model on different varieties of date fruit makes the system able to detect and locate fruit from different varieties accurately. In our case, the dataset contains four famous varieties of dates in Morocco named Majhoul, Boufaguos, Kholt, and Bouisthami.

**Fig. 2:**
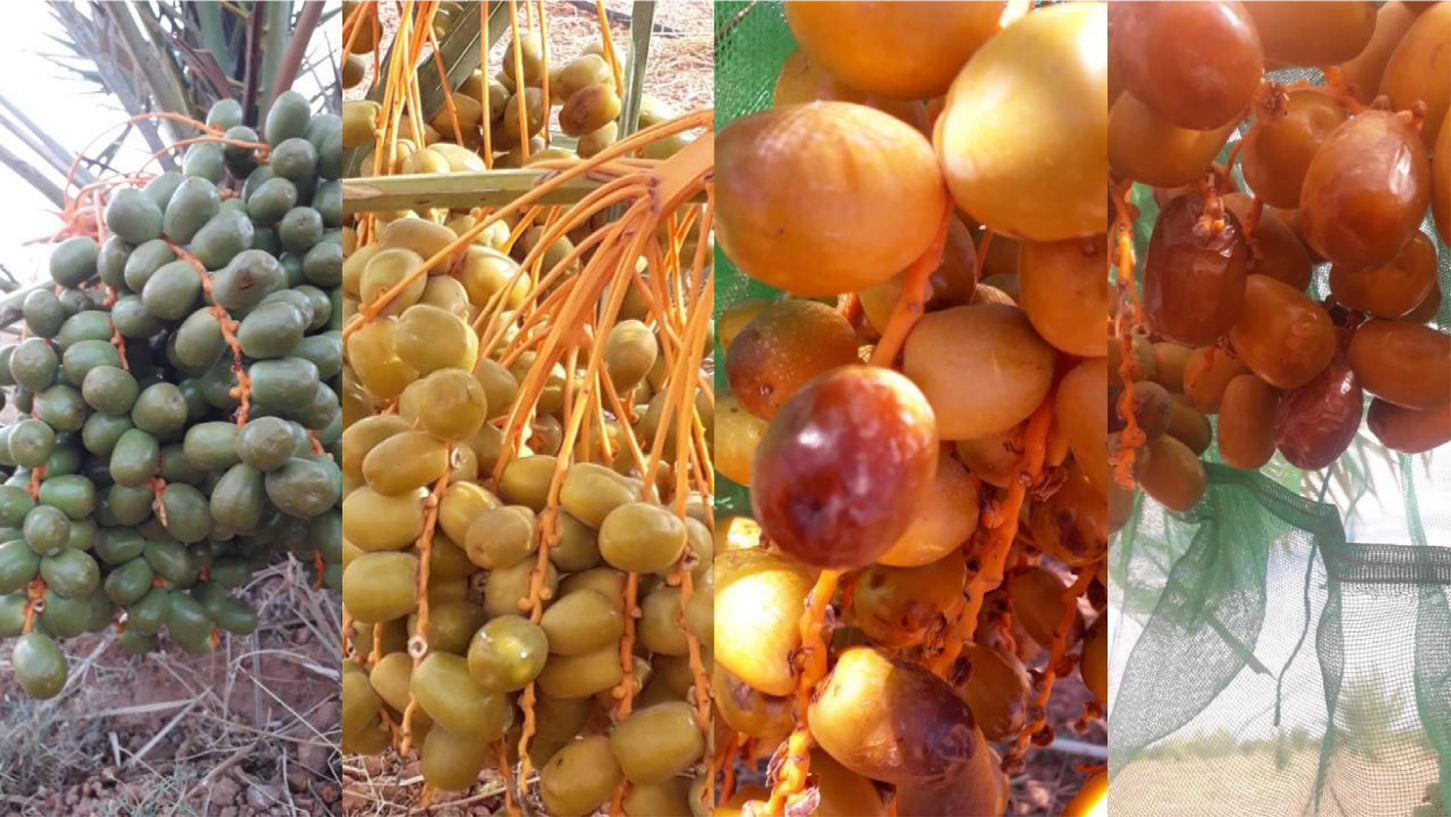
Example of date fruit images at different stages of maturity.

• Date fruit at different stages of maturity:

Date fruit pass through several stages of maturity, each characterized by a specific color, texture, and size. It is essential to train the machine learning model to identify all the maturity stages to ensure that only the right fruit is harvested, considering that harvesting decisions can be in different stages of maturity, as shown in Fig. 2. The presence of dates at different maturity stages within the same cluster poses a challenge in making harvesting decisions. The discrepancy in maturity classes in the annotation file is detailed in Table 2.

• Images of date fruit with various lighting conditions:

**Table 2:** Dataset classification of maturity classes based on degrees of maturity in date bunch.

Maturity class	Date fruit maturity in the bunch
Immature	Bunches > 80% of Immature date fruit
Khalal	Immature <20% and Khalal > 80%
Rutab	Khalal < 20% and Rutab > 80%
Tamar	Rutab < 20% and Tamar > 80%

Lighting conditions can affect the appearance of date fruit, making it a big challenge facing machine vision applications. The dataset contains images taken under all the daylights from 6 am to 6 pm. Therefore, we can train machine learning algorithms that can accurately identify the fruit under all lighting conditions.

• Multi-scale images:

There are two types of date fruit harvesting techniques harvesting whole bunches or single dates. The dataset contains multi-scaled images. Some pictures zoom in on the date fruit that can be used to classify and harvest single dates, and some contains the whole date bunches, taken at close and far distances, which can be used to detect and harvest the whole date bunches.

• Dates covered by bags:

In many regions, the farmer in a specific harvesting session covers date bunches with net mesh bags to protect them from pests and weather conditions, which constitutes a challenge to machine vision to recognize date fruit. Therefore, the dataset contains images of dates in many stages of maturity covered by bags to train the algorithms to locate date bunches even when they are covered. Further, the model can distinguish whether date bunches are bagged or not.

**Fig. 3:**
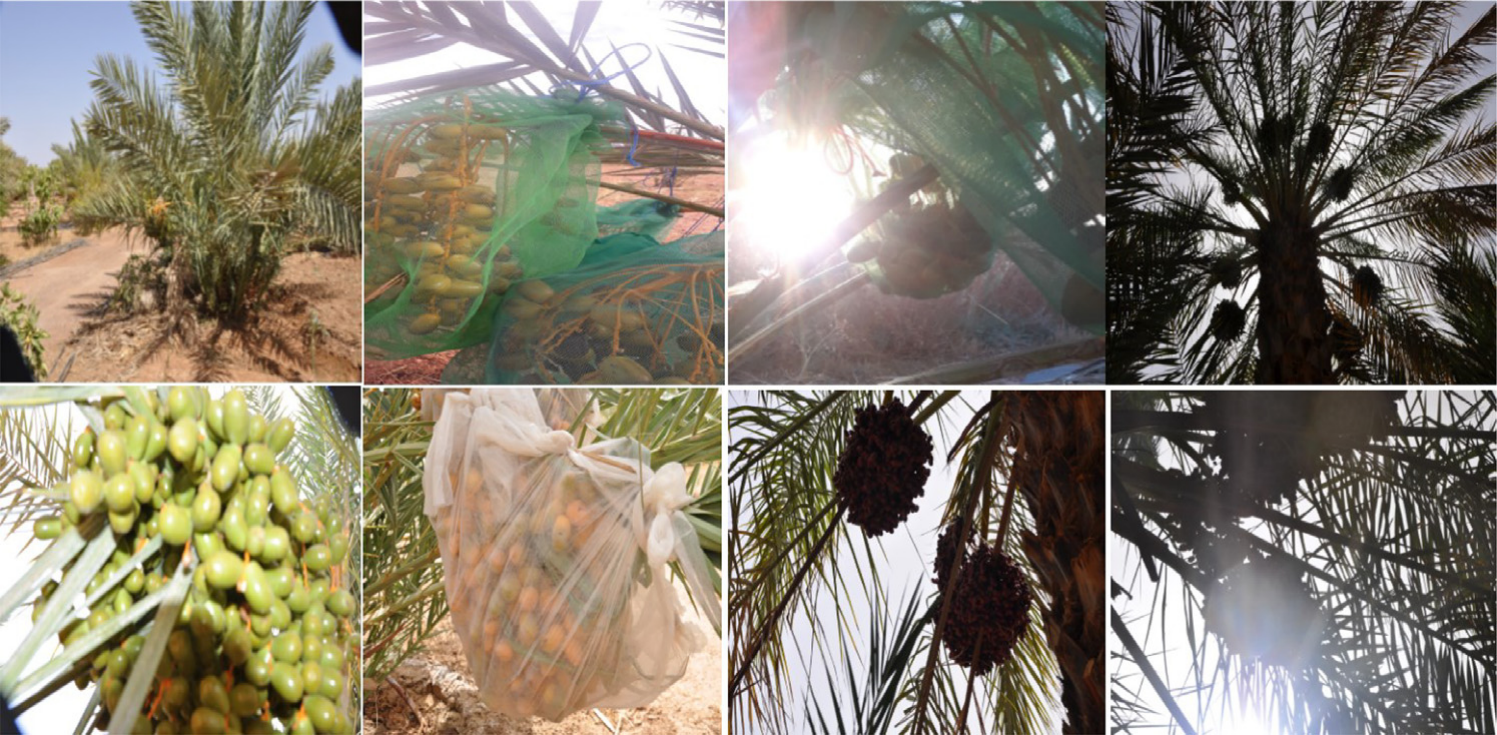
An example of dataset images displaying significant variations in lighting conditions, scales, and dates covered by bags.

Fig. 3 Shows a simple example of dataset images displaying variations in daylight conditions, different scales, and date covered by bags and palms leaves.

## 4 Experimental Design, Materials and Methods

The data were collected in two orchards. The first orchard is located 26km southeast of Errachidia, Morocco (31.8987333, −4.1997700), and the second is in Tismoumine, Alnif Tinghir, Morocco (30.689038927052874, −5.126078230952724). The orchards contain different varieties of dates with palms of different ages and lengths. The dataset contains 9092 images of dates grapes captured with varying scales at different angles from Jun to Sept 2022.

• **Camera specification:**

The data set was collected using two cameras. The first is an RGB smartphone Samsung Ultra camera with a resolution of 2006 × 4128, and the second is a Canon camera with a resolution of 4288 × 2848.

• **Processing:**

**Fig. 4:**
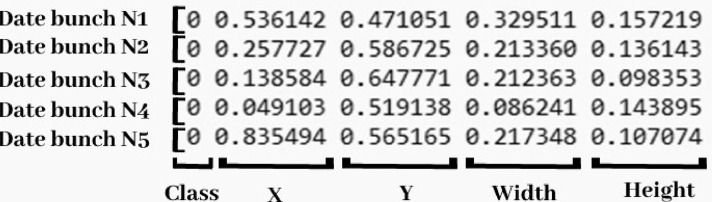
A simple example of a YOLO annotation text file for date detection.

All the dataset images are annotated to detect and locate date fruit using LabelImg software. LabelImg is a tool dedicated to image annotation for object detection by creating a rectangle around the object. The output is a text file with the same name as the input image, containing the object class, *X* and *Y* coordinates, and the height and width of the rectangle, as shown in Fig. 4.

The dataset contains three types of annotations: annotation for date fruit detection, annotation for date fruit varieties, and annotation for date fruit maturity stages. All the annotations file types are text files specific to the You Only Look Once (YOLO) annotation format. Furthermore, the dataset is designed not only for object detection but also includes annotation files for simple classification, enabling the classification of date fruit by date, variety, and maturity stage.

• **General statistics:**

In the dataset, Tables 3 and 4 provide general statistics for each date variety, including the total number of images taken, the total number of date bunches, and the number of palm trees. Table 5 presents the distribution of pictures taken at each maturity stage for every date variety. Table 6 illustrates the use of the percentage of maturity in making harvesting decisions for each variety.

**Table 3:** Number of images, date bunches, and palms in the dataset.

Total number of images	9092
Total number of date bunches	128
Total number of palms	28

**Table 4:** Total number of images, bunches, and palms per date variety.

Date fruit varieties	Total number of images	Total number of bunches	Total number of palms
Boufaguos	4325	50	10
Majhoul	2645	58	14
Bouisthami	360	14	2
Kholt	1762	6	2

**Table 5:** Total number of images in each date fruit maturity stage per variety.

	Immature	Khalal	Rutab	Tamar
**Boufagous**	913	563	1675	1174
**Majhoul**	304	573	1345	423
**Bouisthami**	95	137	46	82
**Kholt**	140	414	649	559

**Table 6:** The percentage of maturity is used to make harvesting decisions for each variety.

	Immature	Khalal	Rutab	Tamar
Boufaguos	Not harvested	Not harvested	Harvested > (30% OF MATURITY)	Harvested
Majhoul	Not harvested	Not harvested	Harvested >(50% OF MATURITY)	Harvested
Bouisthami	Not harvested	Not harvested	Harvested > (70% OF MATURITY)	Harvested
Kholt	Not harvested	Not harvested	Harvested > (60% OF MATURITY)	Harvested

## Limitations

The dataset has certain constraints. While it includes four distinct varieties of date fruits, it does not encompass the entire range of existing date varieties, potentially limiting the model's adaptability to other types of date fruits. Additionally, the dataset does not address seasonal variations in the appearance of date fruits, which may result in performance variability for models trained on it across different seasons.

## Ethics Statement

The production of the data collected did not involve any human subjects, animal experimentation, nor any data from social media platforms. The authors have read and follow the ethical requirements for publication in Data in Brief*.*

## CRediT authorship contribution statement

**Yousra Zarouit:** Conceptualization, Methodology, Investigation, Data curation, Writing – original draft. **Hassan Zekkouri:** Data curation, Writing – review & editing. **Mohamed Ouhda:** Conceptualization, Investigation, Writing – review & editing. **Brahim Aksasse:** Visualization, Supervision, Writing – review & editing.

## Data Availability

Date Fruit Detection Dataset for Computer Vision-Based Automatic Harvesting (Original data) (ZENODO)
